# Transient Global Amnesia Masquerading as Transient Ischemic Attack

**DOI:** 10.7759/cureus.29507

**Published:** 2022-09-23

**Authors:** Cody N Sellers, Rishi Kalia, Keith Gould

**Affiliations:** 1 Neurology, Nova Southeastern University Dr. Kiran C. Patel College Of Osteopathic Medicine, Clearwater, USA; 2 Dr. Kiran C. Patel College of Osteopathic Medicine, Nova Southeastern University, Fort Lauderdale, USA; 3 Emergency Medicine, Nova Southeastern University Dr. Kiran C. Patel College of Osteopathic Medicine, Clearwater, USA

**Keywords:** hypertensive crisis, amnesia, stroke, transient ischemic attack, transient global amnesia

## Abstract

Transient global amnesia (TGA) is a rare self-limited syndrome characterized primarily by anterograde amnesia. It shares multiple characteristics with a transient ischemic attack and ischemic stroke, which carry a more ominous outlook. There is a debate with respect to what is sufficient for ruling out more serious pathologies. Additionally, there is data that challenge the historical view that TGA is a benign condition requiring no long-term management. Here, we present the case of a 70-year-old female who was admitted to a free-standing emergency room with confusion, memory loss, and hypertensive crisis that was diagnosed as TGA. The patient was evaluated by a neurologist and transferred to the hospital. The patient was discharged after more serious pathologies were excluded after an extensive workup. This scenario showcases how physicians have to balance the risk of serious diseases with the need for further testing. Future research should focus on how to accurately identify or rule out serious diseases leading to a reduction in adverse events and patient costs. It is also not clear if TGA is truly benign or has an association with stroke.

## Introduction

Transient global amnesia (TGA) is a rare self-limiting syndrome that is defined as anterograde memory loss that resolves within 24 hours without any long-term damage. Approximately 30-90% of TGA patients report an event that precedes memory loss. These events include both physical stimuli, such as cold water or Valsalva, and emotional stimuli [1]. Patients typically only have one event in their lifetime. Associated symptoms are common and often include nausea, vomiting, and headache [2].

The underlying pathophysiology of TGA is not well understood. Suggested theories include vascular congestion, arterial ischemia, migraine, or an underlying psychosomatic disorder. There is a wide variety of literature with conflicting opinions. The vascular congestion hypothesis is considered the strongest theory due to the frequency of preceding Valsalva [1]. However, evidence from the Korean National Health Insurance Service has shown increased rates of transient ischemic attack (TIA) in those with a history of TGA, which could suggest an arterial ischemia model [3]. If TGA is due to underlying ischemia, it is possible that it may have some relation to ischemic stroke. One facet of the underlying disease process is that the areas in the brain affected include the mediobasal temporal lobe and the hippocampus [1].

TGA is historically considered a benign condition. Once serious pathologies are ruled out, patients can be observed until they return to baseline. Emerging evidence has suggested shared pathophysiology with stroke. It is vital that research focuses on identifying the underlying cause of TGA and determining if further management is warranted.

Patients often present with various symptoms. Often there is overlap between many different disease processes. A crucial step in the evaluation of a patient is differentiating between diseases that need management and those that do not. TGA often presents similar to various conditions, including stroke, hypertensive encephalopathy, seizure, and migraine. If TGA is a benign condition, research should additionally focus on distinguishing it from other conditions.

## Case presentation

A 70-year-old previously healthy female presented to a free-standing emergency room (ER) for a stroke alert with an approximately two-hour history of confusion. The patient’s husband was the primary historian for the patient, and the husband denied any trauma or additional associated symptoms. A review of systems was negative. The patient’s husband denied any significant medical, surgical, social, or family history. On admission, she was oriented only to self. A physical examination revealed a blood pressure of 232/98 mmHg (see Table 1). Her National Institutes of Health (NIH) stroke scale was 2 at the time of admission due to incorrect responses given for the current month and patient age (see Table 2). The attending physician ordered a non-contrast computed tomography (NCCT) of the head to rule out stroke, which was negative. A urinary analysis was ordered to assess for an asymptomatic urinary tract infection because these sometimes cause delirium in older females (see Table 3). The urinalysis was normal. Blood work, which included a complete blood count, comprehensive metabolic panel, and troponin I, was ordered to rule out metabolic abnormalities, infectious etiologies, and myocardial infarction (see Tables 4-6). No abnormalities were found in the blood results. A neurology telehealth consultation was ordered for further assessment of a possible TIA.

**Table 1:** Vital signs of the patient.

Vital sign	Patient value	Reference range
Heart rate (beats per minute)	78	60–100
Blood pressure (mmHg)	232/98	90/60–120/80
Respiratory rate (respirations per minute)	18	12–20
Oxygen saturation (%)	99	93–100
Temperature (°F)	97.8	97.8–99.1

**Table 2:** National Institutes of Health (NIH) stroke scale.

NIH category	Patient score	Reference range
Consciousness	2	0
Commands	0	0
Gaze	0	0
Visual fields	0	0
Facial palsy	0	0
Motor arm	0	0
Motor leg	0	0
Limb ataxia	0	0
Sensory	0	0
Language	0	0
Dysarthria	0	0
Extinction and inattention	0	0

**Table 3:** Urinary analysis findings of the patient.

Laboratory test	Laboratory value	Reference range
Urine color	Straw	Yellow/Straw
Urine appearance	Clear	Clear
Urine protein	Negative	Negative
Urine glucose	Negative	Negative
Urine ketones	Negative	Negative
Urine blood	Negative	Negative
Urine nitrites	Negative	Negative
Urine leukocyte esterase	Negative	Negative

**Table 4:** Complete blood count findings of the patient.

Laboratory test	Laboratory value	Reference range
Hemoglobin (g/dL)	13.8	11.9–14.8
Hematocrit (%)	41.8	35–43
Red blood cell count (×10^6^/µL)	4.78	3.8–5.0
Mean corpuscular volume (fL)	87.4	82.5–98
Mean corpuscular hemoglobin concentration (g/dL)	33	32.5–35.2
Red cell distribution width (%)	13	11.4–13.5
Platelet count (×10^3^/µL)	276	153–361
White blood cell count (×10^3^/µL)	7.45	3.8–10.4

**Table 5:** Comprehensive metabolic panel findings of the patient.

Laboratory test	Laboratory value	Reference range
Sodium (mEq/L)	138	135–145
Potassium (mEq/L)	3.9	3.7–5.2
Chloride (mEq/L)	103	96–106
Carbon dioxide (mEq/L)	27	23–29
Calcium (mg/dL)	9.1	8.5–10.2
Glucose (mg/dL)	161	<140
Blood urea nitrogen (mg/dL)	18	6–20
Creatinine (mg/dL)	1.04	0.6–1.3
Estimate glomerular filtration rate (mL/minute)	54	>60
Alkaline phosphatase (U/L)	86	20–130
Aspartate transaminase (U/L)	19	8–33
Alanine transaminase (U/L)	28	4–36
Total bilirubin (mg/dL)	0.3	0.1–1.2
Total protein (g/dL)	7.2	6.0–8.3
Albumin (g/dL)	3.7	3.4–5.4

**Table 6:** Troponin I high‐sensitivity measurements.

Patient’s value	Reference range
9 ng/L	<14 ng/L

Additionally, the physician ordered 1 L 0.9% NaCl, 20 mg atorvastatin, and 81 mg aspirin. These were given to the patient pre-emptively to treat a possible ischemic stroke. Further, 20 mg of hydralazine was given for a hypertensive emergency, which can increase the risk for adverse cardiovascular events.

The consulting neurologist visited the patient via telehealth and determined that there were no other neurologic deficits. He diagnosed the patient with TGA according to the Hodges & Warlow definition listed in Table 7.

**Table 7:** Hodges & Warlow criteria for transient global amnesia. [4].

Diagnostic criteria for transient global amnesia
a) Attacks must be witnessed and information available from a capable observer who was present for most of the attack
b) There must be clear-cut anterograde amnesia during the attack
c) Clouding of consciousness and loss of personal identity must be absent, and the cognitive impairment limited to amnesia (that is, no aphasia, apraxia, etc.)
d) There should be no accompanying focal neurological symptoms during the attack and no significant neurological signs afterward
e) Epileptic features must be absent
f) Attacks must resolve within 24 hours
g) Patients with recent head injury or active epilepsy (that is, remaining on medication or one seizure in the past two years) are excluded

The neurologist recommended two options to the attending physician. These options included either discharge to home with family or admission to hospital for observation. After discussing options with the patient’s husband, there was a consensus to transfer the patient to the hospital for further inpatient workup and monitoring. This was primarily done to ensure the resolution of the hypertensive emergency and to obtain magnetic resonance imaging (MRI) of the head. In addition, there was a consensus that the husband would be unable to handle the patient at home in her current state.

At approximately 8:36 a.m. the following morning, a repeat neurological examination showed restoration of the patient’s memory. By 9:46 a.m., blood pressure had normalized at 120/60 mmHg. The patient was transferred from the free-standing ER at 10:00 a.m. and was transported to the hospital. This was done the following morning due to a lack of bed availability. At 12:11 p.m., an NCCT of the head and a computed tomography angiogram (CTA) of the head and neck revealed no acute findings. At 12:25 p.m., the attending physician at the hospital ordered a non-invasive transthoracic echocardiogram, which showed no significant findings. Afterward, an MRI and a magnetic resonance angiogram (MRA) of the head were completed, showing no acute intracranial findings. The next morning, the patient was determined to be in stable condition after a follow-up neurology consult and was subsequently discharged to home from the hospital with her husband.

## Discussion

A physician’s choice for the medical management of patients is very important and often forecasts long-term patient outcomes. The first step in deciding initial management is an accurate diagnosis. This can be very difficult in diseases with extensive similarities. In TGA and stroke, some of the factors that may be a barrier to a correct diagnosis include patient presentation, patient demographics, and accuracy of the initial testing.

Patient demographics play an important role in creating a differential diagnosis. The incidence of TGA is estimated to be between 5.2 and 10 in 100,000 individuals under 50 years old. These numbers nearly triple after age 50. The mean age of onset for TGA is between 60 and 65 years old [1]. Similarly, TIA has an incidence that quadruples after age 54. Our patient’s age of 70 fits the most common age bracket for both TGA and stroke. The similarity in the age of onset contributes to the difficulty in distinguishing the two. In comparison to TGA, the overall incidence of TIA is much higher [5]. The incidence of ischemic stroke is also much higher with approximately 795,000 cases per year [6]. This difference in incidence raises significantly more clinical suspicion for stroke. The end result is ambiguity in differentiating TGA from a stroke. A patient who presents with overlapping symptoms may have TGA but would have a higher likelihood of being diagnosed with a stroke.

Initial presenting symptoms have a crucial role in making a correct diagnosis. TGA can present with various non-specific associated symptoms, which include headache, nausea, emesis, paresthesia, and visual dysfunction [2]. These have significant overlap with both TIA and other forms of stroke. The patient in this scenario presented in a hypertensive emergency, a risk factor for stroke. Hypertensive emergencies are relatively common in TGA as well [7]. This further obscures the distinction between TGA and stroke. The defining clinical feature of TGA is anterograde amnesia with other types of memory, such as semantic memory, remaining intact [1]. Amnesia can also be a symptom of a stroke [5]. While stroke often presents with additional symptoms, there are cases of stroke that exist where the only symptom is amnesia. In one case reported by physicians in Germany, a 61-year-old male developed amnesia after a Valsalva-like maneuver and was later diagnosed with a stroke [8]. Based on what is known about the symptomatology of TGA and stroke, diagnosis cannot always be made exclusively from history and physical examination. Further diagnostic imaging can play a supportive role in clarifying the underlying diagnosis.

Hypertensive encephalopathy is also considered in this scenario. However, the lack of neurological symptoms and cerebral edema on imaging makes it less likely.

In this scenario, the patient received an NCCT, the typical initial diagnostic test for patients with amnesia to rule out an underlying brain bleed. Ischemic stroke cannot be excluded by NCCT. MRI is the imaging of choice for ischemia and can be used for patients with a high suspicion for an ischemic stroke, such as hypertensive emergency, headache, or sensory deficits. Individuals diagnosed with TGA who do not have any accompanying risk factors do not typically receive further inpatient workups. This relies on previous data regarding TGA which compares long-term outcomes that include risk for stroke [5].

However, a study published by the Journal of Neurology found a significant number of TGA patients scanned with MRI to have acute ischemic cerebral lesions [9]. These types of lesions are clinically defined as silent infarcts in those without neurologic deficits other than the initially presenting amnesia. Silent infarcts have well-known long-term consequences, including impaired mobility, physical decline, depression, cognitive dysfunction, dementia, and clinical stroke. Silent infarction increases the risk of a future stroke by two to four times [10]. The greater sensitivity of the MRI to pick up silent stroke supports the idea that patients diagnosed with TGA should receive an MRI regardless of additional presenting symptoms (or the lack thereof).

A possible association between silent infarcts and TGA contrasts with the conventional perception that TGA is a benign condition. However, there have been several studies that have shown no difference in the risk of adverse long-term outcomes, including cognitive impairment, seizure, and cerebrovascular events in patients with a history of TGA [11, 12]. One study in Germany examined changes in long-term outcomes with individuals identified with hippocampal CA1 region lesions via MRI with diffusion-weighted imaging (DWI) [13]. Similar to the Journal of Neurology study, this study also found no clinical difference in outcome between those with infarcts and those without.

There are significant discrepancies between the data on TGA and silent stroke. The data mentioned previously suggest an association between the two; therefore, it is logical to suspect similar long-term outcomes. There are various explanations for these differences. One possibility would be multiple underlying causes. Another explanation is that silent infarcts in the hippocampal CA1 area could be the exception regarding long-term outcomes. The hippocampus is considered an area of high neuroplasticity and has an elevated ability to adapt and compensate for damage.

One factor to consider is the potential burden of changing the standard of care for those who do not have a silent infarction. While the long-term financial burden of a stroke in the future is significantly greater, there are costs associated with expensive diagnostic tests such as MRIs. In a report by the Journal of Stroke and Cerebrovascular Disease, the average hospitalization for a stroke ranged from $20,396 to $43,652 [14]. On the other hand, the average cost for an MRI of the brain can cost up to $8,000 [15]. If an MRI would have resulted in the identification of a silent infarct and prevented a future stroke, the benefit is well worth the cost. However, widespread testing for silent infarction would likely lead to a significant burden on patients and the healthcare system. Further research is needed to analyze whether the benefit from additional testing is worth the cost.

## Conclusions

TGA is a rare condition that has historically been considered benign but can present very similarly to more serious pathologies. This case of a 70-year-old female is interesting due to overlapping symptoms and a vague clinical vignette. The initial workup was negative for more serious underlying pathologies, and the patient was managed more conservatively due to the presence of risk factors that increased her risk of stroke. Further research needs to be done to develop more practical and cost-effective methods to accurately recognize TGA patients who may have an underlying stroke. Research should clarify if these infarcts are incidental or related to the TGA. Additionally, the importance of these infarcts to future management should be explored. Doing so has the potential to significantly improve the lives of those affected medically, emotionally, and financially.
